# Supplementary material for: Modeling glioblastoma heterogeneity as a dynamic network of cell states
Source: Mol Syst Biol. 2021 Sep 16;17(9):e10105. doi: 10.15252/msb.202010105 (PMC8444284; doi:10.15252/msb.202010105)
Supplement: Supplementary file 5 — Source Data for Figure 3 [file MSB-17-e10105-s001.zip › Figure3A_sourcedata/GSEA_3065/hallmarks_state5.GseaPreranked.1623416302988/HALLMARK_IL6_JAK_STAT3_SIGNALING.html]

Details for gene set HALLMARK\_IL6\_JAK\_STAT3\_SIGNALING[GSEA]

|  || Dataset | state5 |
| Phenotype | NoPhenotypeAvailable |
| Upregulated in class | na\_pos |
| GeneSet | HALLMARK\_IL6\_JAK\_STAT3\_SIGNALING |
| Enrichment Score (ES) | 0.38880903 |
| Normalized Enrichment Score (NES) | 1.2690626 |
| Nominal p-value | 0.10060976 |
| FDR q-value | 0.14722303 |
| FWER p-Value | 0.604 |
Table: GSEA Results Summary

  

Fig 1: Enrichment plot: HALLMARK\_IL6\_JAK\_STAT3\_SIGNALING      
 Profile of the Running ES Score & Positions of GeneSet Members on the Rank Ordered List

  

| PROBE | GENE SYMBOL | GENE\_TITLE | RANK IN GENE LIST | RANK METRIC SCORE | RUNNING ES | CORE ENRICHMENT || 1 | CD9 |  |  | 14 | 0.857 | 0.1606 | Yes |
| 2 | CD44 |  |  | 378 | 0.349 | 0.1861 | Yes |
| 3 | IFNAR1 |  |  | 614 | 0.282 | 0.2133 | Yes |
| 4 | TNFRSF12A |  |  | 615 | 0.282 | 0.2666 | Yes |
| 5 | IL6ST |  |  | 627 | 0.279 | 0.3182 | Yes |
| 6 | STAT3 |  |  | 797 | 0.242 | 0.3451 | Yes |
| 7 | IFNGR1 |  |  | 983 | 0.213 | 0.3646 | Yes |
| 8 | STAT2 |  |  | 1350 | 0.166 | 0.3552 | Yes |
| 9 | TNFRSF1A |  |  | 1377 | 0.163 | 0.3831 | Yes |
| 10 | OSMR |  |  | 1571 | 0.144 | 0.3888 | Yes |
| 11 | PDGFC |  |  | 1789 | 0.127 | 0.3887 | No |
| 12 | IL13RA1 |  |  | 2016 | 0.110 | 0.3843 | No |
| 13 | CSF1 |  |  | 2643 | 0.071 | 0.3278 | No |
| 14 | IL17RA |  |  | 2656 | 0.071 | 0.3398 | No |
| 15 | IRF1 |  |  | 2926 | 0.056 | 0.3205 | No |
| 16 | TYK2 |  |  | 3436 | 0.030 | 0.2693 | No |
| 17 | PTPN2 |  |  | 3443 | 0.030 | 0.2742 | No |
| 18 | TGFB1 |  |  | 3503 | 0.027 | 0.2728 | No |
| 19 | TNFRSF1B |  |  | 3727 | 0.019 | 0.2515 | No |
| 20 | STAM2 |  |  | 4278 | -0.005 | 0.1910 | No |
| 21 | SOCS3 |  |  | 4805 | -0.026 | 0.1371 | No |
| 22 | CBL |  |  | 4814 | -0.026 | 0.1412 | No |
| 23 | BAK1 |  |  | 5425 | -0.049 | 0.0824 | No |
| 24 | STAT1 |  |  | 6029 | -0.076 | 0.0293 | No |
| 25 | PTPN1 |  |  | 6507 | -0.101 | -0.0047 | No |
| 26 | HMOX1 |  |  | 6561 | -0.106 | 0.0093 | No |
| 27 | MYD88 |  |  | 6788 | -0.120 | 0.0068 | No |
| 28 | IFNGR2 |  |  | 6865 | -0.127 | 0.0223 | No |
| 29 | HAX1 |  |  | 7183 | -0.154 | 0.0160 | No |
| 30 | GRB2 |  |  | 7531 | -0.195 | 0.0141 | No |
| 31 | TNFRSF21 |  |  | 7627 | -0.208 | 0.0427 | No |
| 32 | JUN |  |  | 7930 | -0.262 | 0.0585 | No |
| 33 | PTPN11 |  |  | 8160 | -0.312 | 0.0920 | No |
Table: GSEA details [plain text format]

  

Fig 2: HALLMARK\_IL6\_JAK\_STAT3\_SIGNALING: Random ES distribution      
 Gene set null distribution of ES for **HALLMARK\_IL6\_JAK\_STAT3\_SIGNALING**
